# Using explainable AI to identify disease-relevant and deep brain stimulation treatment-sensitive gait features in Parkinson’s disease

**DOI:** 10.1186/s12984-026-01997-6

**Published:** 2026-04-27

**Authors:** Zhongke Mei, Alain Ryser, Gianluca Amprimo, Jinhao Wang, Julia Vogt, Deepak K. Ravi

**Affiliations:** 1 Laboratory for Movement Biomechanics, Institute for Biomechanics, Department of Health Sciences and Technology, ETH Zürich, Zürich, Switzerland; 2 Medical Data Science, Institute for Machine Learning, Department of Computer Science, ETH Zürich, Zürich, Switzerland; 3 Department of Control and Computer Engineering, Politecnico di Torino, Turin, Italy

**Keywords:** Parkinson’s disease, Deep brain stimulation, Machine learning, Gait characteristics

## Abstract

**Background:**

Gait impairment is a hallmark motor deficit of Parkinson’s disease (PD) and represents an important, yet insufficiently understood, target of subthalamic deep brain stimulation (DBS). Although DBS can improve several motor symptoms, identifying robust and physiologically meaningful gait biomarkers that capture both disease-related deficits and stimulation-induced improvements remains a major challenge. In particular, conventional mean-based gait metrics often fail to fully characterize pathological gait or treatment responsiveness.

**Methods:**

We analyzed 35 spatiotemporal gait parameters obtained during continuous walking from individuals with PD assessed before and after subthalamic DBS, alongside age-matched healthy controls. Multiple machine learning classifiers were evaluated to discriminate between groups, with extreme gradient boosting (XGBoost) achieving the best performance. To enhance interpretability and reduce redundancy among correlated parameters, grouped SHapley Additive exPlanations (SHAP) were applied to rank feature importance and guide feature selection.

**Results:**

Feature selection consistently highlighted step width variability, step width asymmetry, bilateral interlimb coordination, and the anteroposterior margin of stability as the most discriminative parameters. A compact set of five overlapping features after selection not only reliably distinguished PD gait from healthy controls but also demonstrated a shift toward healthy ranges following DBS. Importantly, these selected features outperformed conventional mean-based metrics in capturing both pathological gait characteristics and treatment-related changes.

**Discussion:**

Our findings demonstrate that explainable artificial intelligence approaches can identify physiologically grounded gait features that may serve as candidate markers of both PD severity and DBS responsiveness. By emphasizing variability, asymmetry, coordination, and dynamic stability measures, this approach moves beyond traditional mean-based metrics and provides more sensitive markers of neuromodulation effects. These results support the use of interpretable machine learning frameworks to enable more precise evaluation of DBS outcomes and may contribute to individualized patient management strategies in Parkinson’s disease.

**Supplementary Information:**

The online version contains supplementary material available at 10.1186/s12984-026-01997-6.

## Introduction

 Parkinson’s disease (PD) is a progressive neurodegenerative disorder primarily characterized by the degeneration of dopaminergic neurons within the substantia nigra pars compacta [1]. PD is notably the most common age-related motoric neurodegenerative disease, with prevalence rates reported to range from 108 to 257 per 100,000 individuals in Europe [1, 2]. The motor symptoms of PD are diverse, including tremor, rigidity, bradykinesia, and postural instability [3–5]. Gait disturbances and postural instability are particularly significant challenges in PD, affecting mobility, independence, and quality of life [6–8]. Gait impairments in PD commonly include reduced gait speed and step length, as well as impaired rhythmicity of walking patterns [9, 10], frequently accompanied by asymmetric lower limb movements [11, 12] and reduced bilateral coordination [13, 14]. Levodopa is the gold standard for managing motor symptoms in PD [15]. However, its effects are limited to motor improvement only [16]. Moreover, the effectiveness of the medication may diminish over time with the disease progression, which leads to motor fluctuations and the onset of medication-related complications such as dyskinesia [17–19].

Deep brain stimulation (DBS) has become an essential therapeutic option for addressing motor symptoms in PD, especially for patients experiencing limited benefits from medication, such as those with persistent tremors [20, 21]. This surgical technique involves precisely placing electrodes into targeted regions of the brain, with the subthalamic nucleus (STN-DBS) being a primary target due to its critical role in regulating motor functions [22]. These electrodes deliver high-frequency electrical stimulation, modulating abnormal neural patterns and restoring functional balance in motor circuits [23]. Consequently, STN-DBS provides sustained symptom relief, diminishes motor fluctuations, and significantly enhances patients’ quality of life [24–26].

Even though STN-DBS is well established as an effective treatment for managing motor symptoms in PD, its benefits have been primarily demonstrated through reductions in scores on Part III of the Unified Parkinson’s Disease Rating Scale (UPDRS), which evaluates core motor signs such as tremor, rigidity, and bradykinesia [27–30]. However, gait, a key determinant of functional mobility and independence, is only minimally represented in this clinical assessment. As a result, the specific effects of STN-DBS on gait remain less well understood. This limited representation may lead to an underestimation of gait-related impairments during clinical evaluation and surgical planning, potentially contributing to the variability in treatment outcomes observed among people with PD [31–33].

Recent advancements in gait analysis, including optical motion capture systems and wearable sensors, have allowed quantification of detailed spatiotemporal characteristics of gait [34–36]. Quantitative assessments have highlighted specific alterations due to the PD in gait parameters, such as increased gait variability, increased cadence, reduced stride length, reduced swing duration, and prolonged stance duration, even in early disease stages [8, 36–38]. The quantification of spatiotemporal gait parameters is also employed in evaluating the effectiveness of DBS treatments in PD. Several studies have demonstrated the beneficial effects of STN-DBS on gait parameters, such as increased stride length, improved gait velocity, reduced double-stance duration, and decreased gait variability [39–44]. Conversely, other investigations reported that STN-DBS could exacerbate certain gait impairments, including increased gait asymmetry, reduced gait speed, shorter step lengths, increased cadence, and heightened gait dyscoordination [27, 45–47].

Despite increasing interest in utilizing gait metrics to evaluate PD, their interpretation remains challenging due to the complex and multifactorial nature of gait. Many spatiotemporal gait features are influenced not only by disease severity but also by stimulation settings, compensatory mechanisms, and other individual-specific factors [46, 48, 49]. As a result, analyses that focus on isolated gait metrics may lead to oversimplified interpretations that fail to capture the complex interplay between underlying pathology and neuromodulation-induced changes [50]. To overcome this, there is a critical need to identify a concise and reliable subset of gait biomarkers, defined as parameters that exhibit consistent, reproducible associations with clinical severity or therapeutic response across individuals and measurement contexts. However, this is complicated by redundancy among features, susceptibility to confounding, and the increased noise and computational complexity that arise from high-dimensional datasets. These challenges hinder the extraction of clinically meaningful insights and highlight the importance of reducing redundancy.

In recent years, machine learning (ML) techniques have emerged as powerful tools for navigating the complexity of high-dimensional gait datasets. These data-driven approaches not only enable the objective identification of key gait parameters that best discriminate between Parkinson’s disease (PD) and healthy gait patterns using algorithms such as Random Forest, Support Vector Machine, and Artificial Neural Networks [49, 51–57]. However, with the combination of explainable AI, the data-driven approach will also allow for transparent interpretation of model decisions. XAI frameworks such as SHapley Additive exPlanations (SHAP) provide both global and individual-level quantification of feature contributions, making it possible to link algorithmic outputs to clinically meaningful gait characteristics. This transparency is critical for clinical adoption, as it enables healthcare professionals to understand why a model prioritizes certain parameters, thereby enhancing trust, interpretability, and the potential for integration into decision-support systems. Previous studies applying such approaches have repeatedly identified features such as step length, step width variability, stride length variability, double-support time variability, stance phase duration, and gait asymmetry as discriminative candidate markers for PD, underscoring the value of interpretable methods in highlighting clinically relevant gait metrics [49, 52, 56, 58].

Nevertheless, most existing studies have either focused on differentiating PD gait patterns from those of healthy controls or on predicting surgical outcomes using preoperative assessments alone [59]. As a result, there remains a critical lack of research aimed at systematically identifying gait parameters that are both characteristics of the motor impairments seen in individuals eligible for DBS and sensitive to DBS-treatment-associated changes. This gap limits our ability to establish gait candidate markers that are not only reflective of advanced disease features but also responsive to therapeutic treatment. Addressing this limitation is essential for advancing the development of reliable, objective markers of treatment efficacy and supporting more individualized DBS strategies in clinical practice.

In this study, we address this gap by leveraging quantitative gait data from individuals with PD who underwent STN-DBS. By analyzing gait patterns both before and after surgery and comparing them with data from age-matched healthy controls, we aimed to derive a concise set of candidate gait markers that may support objective evaluation of gait changes relevant to DBS-treated clinical status. Using an explainable AI framework combining machine learning–based classification with SHAP-driven interpretability, we derived candidate gait markers that may support objective evaluation of DBS effectiveness and inform more personalized management strategies in PD.

## Materials and methods

### Data sets description

For this study, we used a previously collected dataset introduced by Mei et al. 2023 [60] and Amprimo et al. 2024 [59]. The dataset comprises 49 individuals diagnosed with Parkinson’s disease (PD), whose gait kinematics were recorded in their best medical state: ON-medication condition prior to STN-DBS electrode implantation (PRE), and in the combined ON-medication and ON-stimulation condition approximately six months post-surgery (POST) [60]. Ethical approval for data collection was granted by the Kantonale Ethikkommission Zurich (Protocol Number: 201500141), and all participants provided written informed consent. As detailed in prior studies using this dataset [27, 60], participants walked barefoot for 10 continuous minutes at a self-selected pace without any form of assistance. The walking task followed an “8”-shaped path marked by two signs placed 10 m apart, allowing for the capture of multiple consecutive gait cycles in a controlled laboratory environment. We only used the straight-walking segments of the trials for our analysis. Compared to similar studies, a longer trial duration was intentionally chosen to improve the accuracy and reliability of both the mean and variability measures of gait parameters [61]. Although the extended duration could have induced fatigue, no participants reported feeling fatigued or requiring breaks between trials. Gait data were recorded using a three-dimensional motion capture system (Vicon Nexus, version 2.3/2.8.2, Oxford Metrics, UK), consisting of 10 cameras and 61 reflective markers, sampled at 100 Hz.

### Gait parameter calculations

Gait parameters were computed using custom MATLAB scripts (version R2022a, The MathWorks Inc., Natick). In total, we selected 35 gait parameters, including the mean, variability, and asymmetry of common spatiotemporal parameters, along with measures of upper and lower limb coordination and margin of stability. The common spatiotemporal parameters include cadence (Cadence), double limb stance time (DLST), stride time (StrideT), stance time (StanceT), swing time (SwingT), step time (StepT), step length (StepL), step width (StepW), stride length (StrideL), and walking speed (WalkingSpeed). We computed the mean value of each common spatiotemporal parameter by averaging all individual steps recorded during the 10-minute walking trial. Asymmetry value (Asy) was evaluated for step length (StepL_Asy), step width (StepW_Asy), step time (StepT_Asy), swing time (SwingT_Asy), and stance time (StanceT_Asy) [62]. Coordination between limbs was assessed using two established approaches: the Phase Coordination Index (PCI) [63] and Continuous Relative Phase (CRP) [13, 14]. PCI quantifies the consistency and accuracy of interlimb timing and was calculated for left–right step coordination (PCI_LeftvsRight) and for coordination between short and long gait cycles (PCI_ShortvsLong). CRP was used to assess the relative timing between limb segments and included coordination between upper limbs (CRP_arm&arm), lower limbs (CRP_Leg&Leg), and combined upper–lower limb interactions (CRP_Rarm&Lleg, CRP_Larm&Rleg, CRP_Larm&Lleg, CRP_Rarm&Rleg). The margin of stability was defined as the distance between the extrapolated center of mass and the base of support and was evaluated separately in the anteroposterior and medio-lateral directions [64–66]. The variability (Var) of each spatiotemporal parameter was quantified using the coefficient of variation, defined as (standard deviation/mean) × 100. The specific definition of each parameter is provided in Supplementary Methods 1. For common spatiotemporal parameters, foot clearance, and margin of stability, values from left and right gait cycles were averaged. Asymmetry and coordination parameters were computed for each gait cycle and then averaged over the whole trial after removing outliers, defined as values exceeding ± 4 median absolute deviation (MAD) from the median.

To investigate group-level effects on individual gait parameters, we performed independent-samples t-tests for comparisons between PRE and age-matched healthy controls and paired t-tests for comparisons between PRE and POST. Because 35 gait parameters were tested within each comparison family, we applied the Benjamini–Hochberg false discovery rate (FDR) procedure separately to the PRE-control and PRE-POST analyses. We report unadjusted p-values for transparency, while FDR-adjusted q-values, effect sizes (Hedges’ g), and 95% confidence intervals are provided in Supplementary Table 1. Given the correlation structure among gait parameters and the fact that these univariate analyses were intended to complement rather than replace the multivariate machine-learning framework, they were interpreted as supportive and exploratory.

### Classification framework

**Fig. 1:**
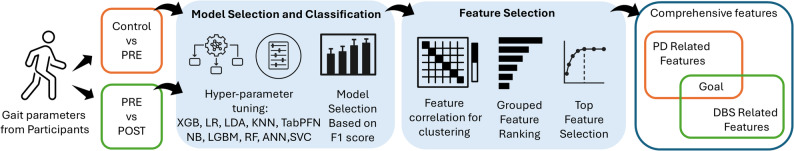
Overview of the gait-based classification and feature selection pipeline. Gait parameters were used to classify control vs. PRE and PRE vs. POST conditions. Multiple machine learning models were tuned and evaluated based on the F1 score. Feature selection included correlation-based grouping and importance ranking. Final features were categorized into PD-related and DBS-related subsets, with the overlap, as the final goal, between the two highlighting candidate gait markers that are both disease-relevant and treatment-responsive

The classification framework comprised two primary objectives: first, to distinguish between PD patients and healthy controls; and second, to differentiate gait characteristics of patients before (PRE) and after (POST) DBS surgery. An identical workflow, comprising preprocessing, feature selection, classification, and evaluation, was applied to each classification objective. These steps are illustrated schematically in Fig. 1.

For the first classification objective, we used a cohort of 49 PwPD and 51 healthy controls. To ensure comparability between groups, we performed one-to-one nearest-neighbor matching based on age using the R package *MatchIt* [67]. Specifically, each patient was matched to a healthy control within a caliper of 0.5, effectively controlling for age-related confounding in our analysis.

After matching, we confirmed that there was no significant difference in age distribution between groups by performing a t-test with a significance level of α = 0.05. This resulted in a final dataset of 44 PwPD and 44 age-matched healthy controls, which were combined and labeled accordingly. The data were then split into a training set (80%) and a test set (20%). The test set remained untouched throughout the model development and was only used in the final step to evaluate the optimized model with the selected features. Both the training and test sets were standardized separately using the mean and standard deviation of each feature. An extensive machine learning (ML) framework was implemented to systematically select, train, and evaluate several candidate models, as illustrated in Fig. 1. Specifically, we included models previously demonstrated to be effective for similar classification tasks [49, 56, 57, 68–70], such as XGBoost (XGB), logistic regression (LR), linear discriminant analysis (LDA), k-nearest neighbor (KNN), Naive Bayes (NB), support vector machine (SVC), random forest (RF), Light Gradient Boosting Machine (LGBM), tabular foundation model (TabPFN) and artificial neural networks (ANN).

For each ML model, hyperparameter tuning was performed using grid search optimization on the training data. Specifically, a 5-fold cross-validation approach was employed, in which the training data were randomly divided into five stratified subsets to maintain class balance in each fold. During cross-validation, each hyperparameter combination was evaluated by training on four folds and validating on the remaining fold, and the hyperparameter set yielding the highest mean validation F1-score across the five folds was selected as optimal. The model with the highest classification performance among the ten optimized models, determined primarily by the mean validation F1-score, was then selected as the best-performing model. Sensitivity, specificity, and AUC of the selected model were also calculated and reported. To reduce the risk of optimistic model evaluation, all model development steps were performed using the training set, whereas an independent hold-out test set was separated before model development and reserved exclusively for final evaluation. Feature importances were extracted directly from the optimized best-performing model, enabling the identification and ranking of the gait parameters that most strongly influenced classification outcomes. In addition, repeated cross-validation using 10 different random seeds and the updated best hyperparameters was performed to assess the stability of model ranking and validation performance across alternative data partitions (Supplementary Fig. 1). This procedure was not implemented as a fully nested cross-validation design with an outer resampling loop; rather, cross-validation was used for model selection within the training set, whereas final generalization performance was assessed on the untouched hold-out test set.

In addition to distinguishing patients from healthy controls, we trained another set of models to differentiate between patients’ gait characteristics before and after DBS surgery. The same modelling framework was applied, with the additional constraint that PRE and POST samples from the same patient were assigned exclusively to either the training set or the hold-out test set, thereby preventing data leakage between model development and final evaluation. The optimized model derived from the training data was then applied to the untouched test set to assess its final internal generalization performance.

### Explainable AI-based gait feature selection

To identify the most critical gait parameters that differentiate PwPD from healthy controls, we employed an explainable AI approach using SHAP values to quantify feature contributions in a model-agnostic manner. In addition to the model’s built-in importance metrics, we employed SHAP values to gain deeper insights into feature contributions. SHAP is a consistent, model-agnostic interpretive framework that quantifies the marginal contribution of each feature to the model’s predictions, facilitating both global and individual-level explanations [71].

Before computing SHAP values, we evaluated Pearson’s correlation between individual features to reduce the impact of multicollinearity on model interpretability. Features with correlation coefficients greater than 0.9 were grouped together, whereas features below this threshold remained separate. This threshold was intentionally set high so that only near-redundant features were merged, thereby limiting unnecessary loss of feature-level interpretability while still reducing instability in importance attribution caused by highly correlated inputs. SHAP values were then calculated at the group level to estimate the overall contribution of each correlated feature cluster to the classification process. This grouping strategy was used to guide feature ranking, while the final selected parameters were still reported and interpreted individually where possible. These grouped SHAP rankings were used to guide the subsequent iterative feature selection. We then adopted an iterative feature selection process, incrementally adding the top-ranked parameters and evaluating model performance at each step. At each iteration, we computed cross-validated F1-scores and identified the subset of gait parameters that yielded the highest classification accuracy. If multiple parameter subsets resulted in equally high accuracy, the smallest subset was selected as the optimal discriminative set. Finally, the entire training set was used to train the optimized model with the selected features, which was then applied to classify the test set. To further reduce optimism in feature selection, the final selected feature subsets were additionally evaluated on the untouched hold-out test set that had been excluded from model fitting, hyperparameter tuning, and feature ranking. As no independent external cohort was available, this procedure was regarded as internal validation rather than external validation. Classification performance was evaluated using the F1 score, sensitivity, specificity, and AUC. The same methodology was applied to the second classification scenario, which focused on differentiating gait performance before and after DBS surgery.

## Results

### Participant demographics

The original cohort comprised 51 healthy controls, who were each evaluated once, and 49 PwPD evaluated both PRE and POST surgery. After age-matching, 44 PwPD in the PRE condition were matched with 44 healthy controls, resulting in no statistically significant age difference between groups (*P* = 0.07). After selection, the average age value for PD is 60.50 (standard deviation: 10.90), while for healthy controls, it is 64.57 (standard deviation: 10.16). Notably, none of the participants exhibited freezing of gait during objective gait assessments; however, mild freezing of gait was self-reported by three participants prior to surgery and five participants following surgery, as indicated by their UPDRS scores. The details of the demographics include mean and standard deviation for age, weight, height, age at disease onset, disease duration, and disease severity according to the Hoehn and Yahr scale, as well as sex distribution, are shown in Supplementary Table 2. Because matching was performed on age only, residual demographic imbalance, including possible differences in sex distribution, cannot be excluded and should be considered when interpreting between-group comparisons.

**Table 1:** The means (SD) of gait parameters for pre-DBS, post-DBS, and the control group are depicted, accompanied by p-values

Gait parameters	Pre-DBS (*N* = 49)	Post-DBS (*N* = 49)	*p*-value (pre-post)	Selected pre-DBS (*N* = 44)	Selected healthy control (*N* = 44)	*p*-value (pre-control)
Cadence (steps/min)	113.46 (7.74)	115.8 (6.81)	**0.004**	113.29 (7.66)	116.64 (8.26)	0.059
DLST (s)	0.13 (0.02)	0.12 (0.02)	0.255	0.13 (0.02)	0.12 (0.02)	0.358
StrideT (s)	1.06 (0.07)	1.04 (0.06)	**0.004**	1.06 (0.07)	1.03 (0.08)	0.065
StanceT (s)	0.66 (0.05)	0.64 (0.05)	0.014	0.66 (0.05)	0.64 (0.05)	0.107
SwingT (s)	0.41 (0.02)	0.4 (0.02)	**0.005**	0.41 (0.02)	0.39 (0.02)	0.035
StepL (cm)	64.49 (8.17)	63.51 (8.28)	0.214	64.93 (7.95)	64.39 (5.48)	0.702
StepT (s)	0.53 (0.04)	0.52 (0.03)	**0.003**	0.53 (0.04)	0.52 (0.04)	0.064
StepW (cm)	7.76 (2.95)	8.19 (3.09)	0.094	7.77 (2.97)	8.16 (2.06)	0.528
StrideL (cm)	127.82 (16.51)	125.77 (16.65)	0.196	128.71 (16.02)	127.6 (11.14)	0.697
Walking speed (cm/s)	120.41 (19.07)	121.29 (18.81)	0.628	120.93 (18.47)	123.88 (13.52)	0.315
StepL Asy	4.98 (3.17)	5.47 (3.14)	0.326	4.81 (3.04)	3.01 (2.12)	**0.001**
StepW Asy	26.95 (11.47)	23.01 (9.45)	**0.005**	27.37 (11.68)	20.94 (6.26)	**0.004**
StepT Asy	3.73 (2.63)	4.4 (2.52)	0.087	3.26 (1.41)	2.53 (1.32)	**0.015**
StanceT Asy	2.72 (2.49)	3.1 (2.21)	0.14	2.36 (1.31)	1.91 (1.0)	0.099
SwingT Asy	3.89 (3.82)	4.5 (3.4)	0.128	3.36 (2.31)	2.75 (1.61)	0.174
PCI_Left vs Right	181.21 (3.9)	180.67 (4.43)	0.385	180.5 (2.88)	180.35 (2.29)	0.798
PCI_ShortvsLong	181.02 (3.98)	180.47 (4.43)	0.38	180.3 (2.91)	180.17 (2.28)	0.817
CRP_Leg&Leg	0.87 (0.21)	0.87 (0.23)	0.894	0.87 (0.25)	0.76 (0.17)	**0.013**
CRP_arm&arm	0.88 (0.21)	0.88 (0.23)	1	0.88 (0.24)	0.77 (0.17)	**0.008**
CRP_Rarm&Lleg	0.84 (0.26)	0.73 (0.1)	**0.003**	0.84 (0.23)	0.69 (0.08)	**< 0.001**
CRP_Larm&Rleg	0.87 (0.25)	0.75 (0.1)	**0.003**	0.87 (0.23)	0.72 (0.08)	**< 0.001**
CRP_Rarm&Rleg	2.63 (0.24)	2.55 (0.08)	**0.018**	2.61 (0.23)	2.53 (0.08)	**0.037**
CRP_Larm&Lleg	2.61 (0.24)	2.53 (0.08)	**0.018**	2.59 (0.23)	2.51 (0.08)	**0.037**
MOS_AP	-29.2 (27.01)	-22.52 (14.25)	0.142	-31.34 (26.47)	-17.84 (16.83)	**0.007**
MOS_ML	69.61 (25.5)	68.48 (22.02)	0.810	71.11 (24.45)	76.8 (19.05)	0.196
Cadence_Var (% CV)	1.64 (0.68)	1.82 (0.86)	0.179	1.57 (0.57)	1.54 (0.86)	0.871
DLST_Var (% CV)	8.29 (2.39)	9.26 (3.34)	0.067	8.08 (2.14)	6.75 (2.59)	**0.018**
StrideT_Var (% CV)	1.96 (0.61)	2.15 (0.82)	0.141	1.89 (0.52)	1.82 (0.86)	0.641
StanceT_Var (% CV)	2.7 (0.74)	3.01 (1.02)	**0.042**	2.6 (0.62)	2.42 (1.06)	0.367
SwingT_Var (% CV)	2.54 (0.68)	2.78 (1.02)	0.096	2.49 (0.66)	2.03 (0.78)	**0.006**
StepL_Var (% CV)	3.52 (1.05)	3.76 (1.31)	0.177	3.4 (0.97)	2.59 (0.64)	**< 0.001**
StepT_Var (% CV)	2.44 (0.59)	2.64 (0.91)	0.134	2.37 (0.51)	2.16 (0.8)	0.167
StepW_Var (% CV)	32.69 (15.3)	28.3 (12.97)	**0.017**	33.24 (15.56)	23.54 (7.74)	**0.002**
StrideL_Var (% CV)	3.11 (1.07)	3.32 (1.27)	0.222	2.96 (0.96)	2.32 (0.73)	**0.002**
WalkingSpeed_Var (% CV)	4.0 (2.67)	3.91 (1.49)	0.816	3.87 (2.61)	3.15 (1.46)	0.128

Statistically significant differences are denoted with bolded numbers

### Comparative analysis of gait parameters across PD and DBS states

The average and standard deviation values of all 35 gait parameters for each group, as well as the P values from the t-tests, are shown in Table 1. To account for multiple testing, Benjamini–Hochberg FDR correction was applied separately to the PRE–control and PRE–POST comparisons (Supplementary Table 1). By comparing PwPD and healthy conditions, 16 out of 35 gait characteristics were significantly affected by PD impairment, and 13 remained significant after FDR correction. These include SwingT, StepL_Asy, StepW_Asy, StepT_Asy, CRP_Leg&Leg, CRP_arm&arm, CRP_Rarm&Lleg, CRP_Larm&Rleg, CRP_Rarm&Rleg, CRP_Larm&Lleg, MOS_AP, DLST_Var, SwingT_Var, StepL_Var, StepW_Var, and StrideL_Var. On the other hand, for the comparison between PRE and POST conditions, 12 out of 35 gait characteristics showed a significant difference, and 7 remained significant after FDR correction. These include Cadence, StrideT, StanceT, SwingT, StepT, StepW_Asy, CRP Rarm&Lleg, CRP Larm&Rleg, CRP Rarm&Rleg, CRP Larm&Lleg, StanceT Var, and StepW Var. Effect sizes and 95% confidence intervals are reported in Supplementary Tables 1 to provide information on the magnitude and precision of these parameter-wise differences.

### Classification performance

**Fig. 2:**
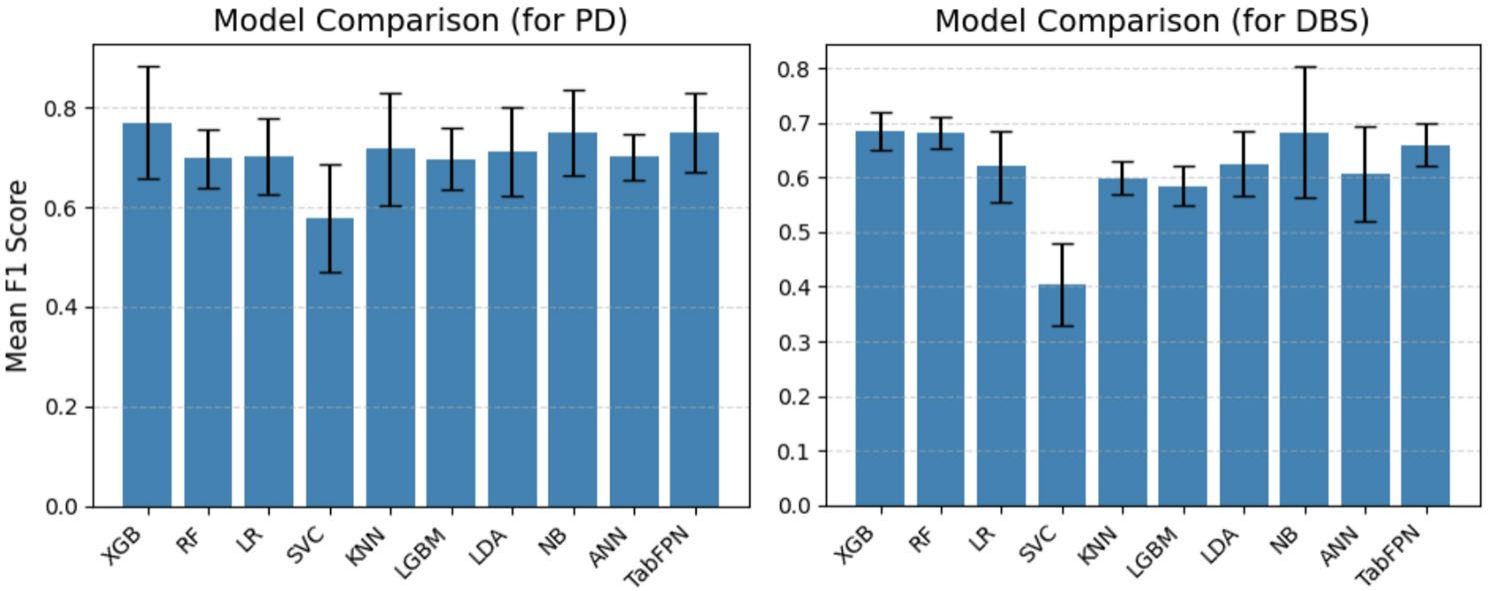
Performance of the candidate models for the two classification tasks: distinguishing PwPD from healthy controls (left) and distinguishing PRE from POST gait in PwPD (right). Bars show the mean ± standard deviation of the validation F1-score across 5-fold cross-validation. F1-score was used as the primary model-selection metric, whereas sensitivity, specificity, and AUC of the selected model are additionally reported in the text

Classification performance was evaluated primarily using the F1-score, while sensitivity, specificity, and AUC were additionally reported for the selected model to provide a broader interpretation of discrimination performance. Figure 2 presents the cross-validated F1-scores on the validation set for the 10 candidate models in both classification scenarios. In the primary analysis, XGBoost yielded the highest validation performance and was therefore selected as the main model for subsequent parameter selection and interpretation. For the classification between healthy controls and PwPD before DBS treatment, the F1 score for the training set was 0.92 ± 0.03 (sensitivity = 0.94 ± 0.05, specificity = 0.89 ± 0.02, AUC = 0.94 ± 0.02), while the F1 score for the validation set was 0.76 ± 0.11 (sensitivity = 0.72 ± 0.10, specificity = 0.80 ± 0.11, AUC = 0.82 ± 0.05; see Fig. 2). For the classification between PRE and POST, the F1 score for the training set was 0.85 ± 0.06 (sensitivity = 0.88 ± 0.05, specificity = 0.82 ± 0.08, AUC = 0.88 ± 0.04), while the F1 score for the validation set was 0.69 ± 0.06 (sensitivity = 0.64 ± 0.04, specificity = 0.73 ± 0.07, AUC = 0.75 ± 0.05). As expected for a dataset of this size, training performance exceeded validation performance in both classification tasks, indicating residual overfitting. To further examine the robustness of model ranking, we repeated the model comparison across 10 different random seeds (Supplementary Fig. 1). In the PD classification task, XGBoost remained among the top-performing models, while TabPFN showed comparable mean validation performance. In the DBS classification task, XGBoost, Naive Bayes, and TabPFN showed similar average validation performance across repeated resampling. We therefore interpret the performance advantage of XGBoost as modest but sufficiently consistent to support its use as the primary explanatory model. Given the comparable predictive performance of TabPFN and the interpretability-focused aim of the present study, XGBoost was retained as the main model for feature ranking and SHAP-based interpretation (Table 2).

**Table 2:** The best-performing hyperparameters for the XGBoost model

Parameters	Healthy controls vs. PRE-DBS	PRE-DBS vs. POST-DBS
Colsample_bytree	0.8	0.8
Gamma	1	1
Learning_rate	0.1	0.0001
Max_depth	3	4
Min_child_weight	1	1
n_estimators	50	150
Reg_alpha	0.1	0.1
Reg_lambda	0.1	10
Subsample	1	0.9

**Fig. 3:**
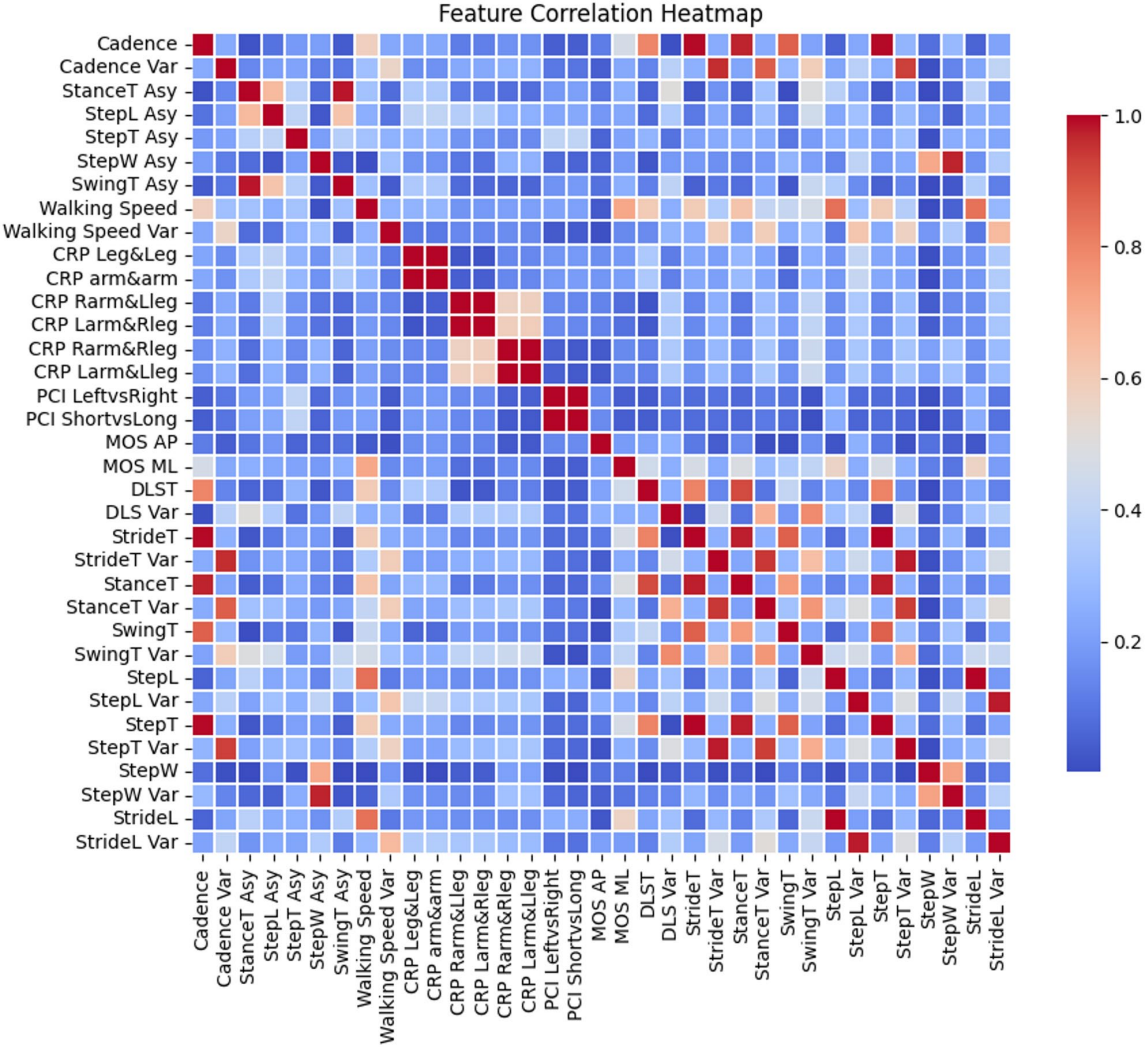
The heatmap shows the correlations across the 35 features. The values represent the absolute value of the Pearson correlation coefficient. Dark blue indicates no correlation (correlation = 0), while dark red indicates perfect correlation (correlation = 1), meaning the two features vary identically

Several of the selected gait features exhibited strong pairwise correlations (Fig. 3). As expected, several features showed strong pairwise correlations, with correlation coefficients approaching 1.0. Notably, features representing different statistical descriptors of the same gait metric, such as mean and variability, or mean and asymmetry, tended to cluster together with high correlations. Similarly, parameters describing left–right and cross-limb coordination (e.g., CRP groups) also demonstrated strong intercorrelations. To address this redundancy and improve interpretability, we grouped together features with correlation coefficients greater than 0.9, resulting in 20 feature clusters. These clusters were subsequently used to compute grouped SHAP values, with detailed information provided in Supplementary Table 3.

**Fig. 4:**
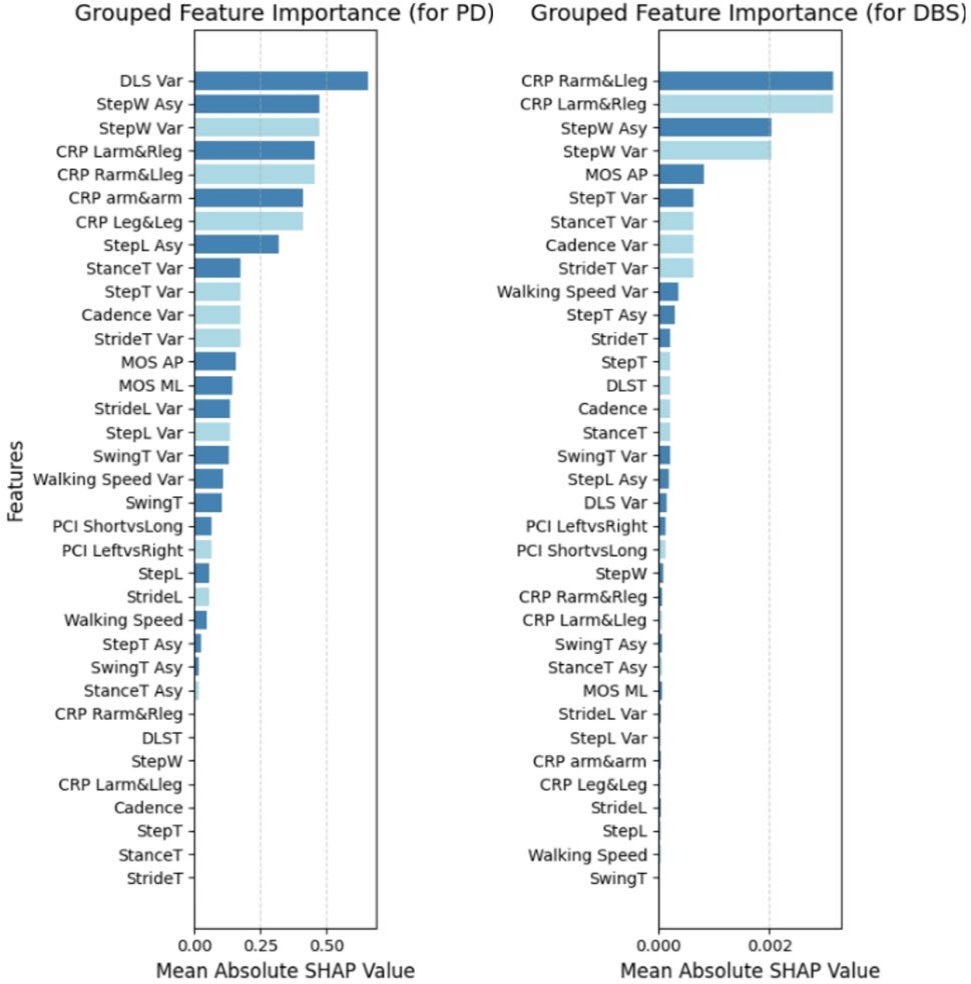
The feature importance ranking of all 35 gait features for PD (left) and DBS (right), which represents the importance of the feature to understand PD or DBS-related effects on patients. Feature importance is represented by the mean absolute SHAP values; higher SHAP values indicate greater influence on the model’s predictions. Features within the same correlation group share the same SHAP value, with the most representative feature shown in dark blue and the remaining group members in light blue. Features are ranked from top to bottom in order of decreasing importance. The top-ranking features are the most important for understanding PD or DBS-related effects on patients

The explainable AI analysis based on grouped SHAP values revealed the grouped feature importance rankings, quantified as mean absolute SHAP values, for the PD versus healthy control classification (Fig. 4 left) and the pre- versus post-DBS classification (Fig. 4 right). In the grouped SHAP plots, dark blue bars represent the most representative feature within each correlation cluster, while the adjacent light blue bars indicate other features within the same cluster (e.g., StepW Asy and StepW Var). In the PD classification task, gait variability and asymmetry emerged as the most discriminative domains, led by double limb stance time variability (DLS Var), step width asymmetry (StepW Asy), and step width variability (StepW Var), followed by bilateral coordination measures such as CRP Larm&Rleg, CRP Rarm&Lleg, CRP arm&arm, and CRP Leg&Leg. In contrast, the DBS classification prioritized cross-limb coordination (CRP Rarm&Lleg, CRP Larm&Rleg), step width asymmetry, step width variability, and anteroposterior margin of stability (MOS AP). Notably, five features, StepW Asy, StepW Var, CRP Larm&Rleg, CRP Rarm&Lleg, and MOS AP, were consistently ranked among the most important in both tasks.

### Parameter selection based on model performance

**Fig. 5:**
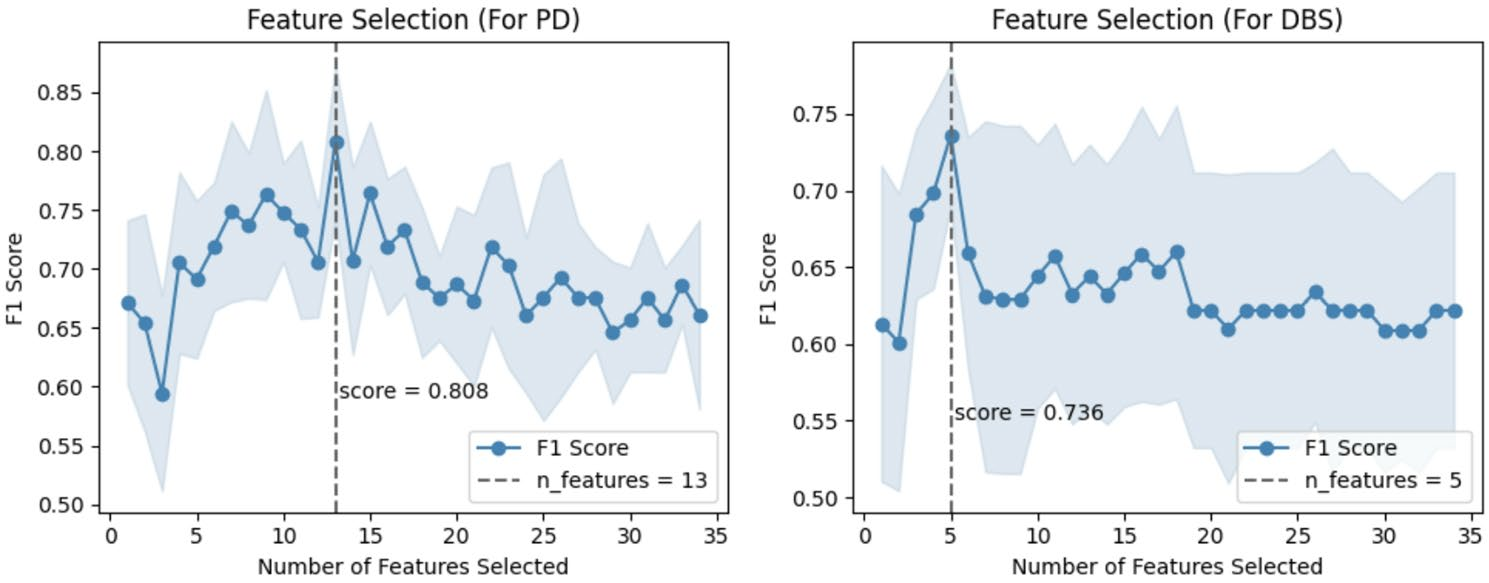
The model’s performance when selecting a different number of the most related gait parameters as the input for PD (up) and DBS (down). The x-axis represents the number of top-ranked features used as input, while the y-axis shows the average F1 score across 5-fold cross-validation (mean ± standard deviation). The dashed line marks the iteration where the model achieved its peak performance, indicating the optimal number of gait features for characterizing the effects of PD and DBS, respectively

We assessed the F1-score while incrementally including top-ranked parameters to identify the gait parameters most influenced by PD and DBS treatment. Figure 5 depicts the relationship between classification performance (F1 score) and the number of selected parameters. For the PD-related classification (PwPD vs. healthy controls), the classifier achieved its highest performance (F1 score: 0.81 ± 0.07, sensitivity = 0.83 ± 0.06, specificity = 0.79 ± 0.09, AUC = 0.84 ± 0.05) when utilizing the top 13 parameters: DLS_Var, StepW_Asy, StepW_Var, CRP_Larm&Rleg, CRP_Rarm&Lleg, CRP_arm&arm, CRP_leg&leg, StepL_Asy, StanceT_Var, StepT_Var, Cadence_Var, StrideT_Var, and MOS_AP. The performance of the optimized model with selected features on classifying the test set is similar (F1 score = 0.75, sensitivity = 0.6, specificity = 1.0, AUC = 0.8). When differentiating gait before and after DBS surgery, the classifier reached optimal performance (F1 score: 0.74 ± 0.05, sensitivity = 0.71 ± 0.04, specificity = 0.76 ± 0.05, AUC = 0.77 ± 0.05) using the top 5 parameters: CRP_Rarm&Lleg, CRP_Larm&Rleg, StepW_Asy, StepW_Var, and MOS_AP. The performance of the optimized model with selected features on the test set using these selected features was similar (F1 score = 0.72, sensitivity = 0.8, specificity = 0.6, AUC = 0.76). The comparable performance observed on the independent hold-out test set suggests that the discriminatory value of the selected feature subsets was not restricted to the cross-validation partitions, although these findings still represent internal rather than external validation.

## Discussion

This study integrated statistical analysis, machine learning, and explainable AI to identify gait parameters that distinguish PD from healthy controls and pre- from post-DBS conditions. Statistical tests revealed group differences, while model-based ranking and selection highlighted parameters with optimized predictive value. Grouped SHAP analysis provided biomechanically interpretable explanations, resulting in feature sets that capture both distinct and overlapping gait characteristics associated with disease and stimulation effects. By systematically identifying gait parameters that both characterize PD-related deficits and respond to DBS, this study addresses a critical gap in the field and provides a framework for linking biomechanical markers with therapeutic outcomes.

### The influence of PD and DBS on gait parameters

Interpretation of the PRE-POST comparison requires caution, because postoperative assessments were performed in the combined ON-medication and ON-stimulation condition. Accordingly, the observed differences should not be interpreted as the isolated physiological effects of stimulation alone. However, the present design remains clinically relevant because DBS is typically not evaluated in isolation in routine practice, but in relation to the patient’s optimized post-surgical treatment state, which reflects the combined effects of stimulation settings, medication status, and clinical programming. From this perspective, the identified gait parameters may be more appropriately interpreted as candidate markers of gait status under optimized DBS-based treatment, rather than as markers of the isolated effect of DBS alone.

Statistical comparisons revealed that medio-lateral stability measures, particularly step width variability and step width asymmetry, were significantly higher in PD than in healthy controls. These abnormalities are consistent with impaired lateral balance control caused by deficits in proprioceptive feedback, as well as asymmetrical motor output that reflects the unilateral onset and progression of PD [72, 73]. Temporal variability measures, including double-support time and stance phase variability, were also elevated, indicating reduced consistency in gait cycle timing and diminished rhythmic motor control by basal ganglia cortical circuits [74–76].

Following DBS, reductions in step width variability and double-support time variability indicated partial restoration of stability control and rhythmicity [44]. Improvements in these parameters are consistent with evidence that subthalamic stimulation can modulate subcortical–cortical and brainstem pathways involved in postural control [39, 48, 77]. However, step width asymmetry persisted after DBS, suggesting that symmetry-related control mechanisms, which are thought to be less responsive to dopaminergic or stimulation-based modulation, remain impaired. This aligns with reports that axial symptoms are resistant to current pharmacological and surgical interventions in PD [78]. Beyond their established role as fall-risk indicators, our results also suggest that step width metrics are sensitive to DBS-related improvements, highlighting their potential dual role as markers of both disease burden and therapeutic efficacy.

### Explainable AI framework and model choice

Ten classifiers from different methodological families were evaluated to avoid bias toward a single modeling approach and to compare their suitability for structured gait data. XGBoost achieved the highest cross-validated F1-scores on the validation set for both classification tasks. This aligns with findings from other structured biomedical datasets, where gradient boosting often outperforms linear models and kernel-based methods [79]. Its advantage here is likely due to its ability to capture nonlinear relationships among heterogeneous gait parameters, built-in regularization through L1/L2 penalties, and robustness to skewed or interdependent variables [80].

Although hyperparameter tuning and regularization were used to improve generalization, the gap between training and validation performance indicates residual overfitting, most likely due to the limited sample size relative to the dimensionality of the gait feature space [81]. To address this more explicitly, we added repeated cross-validation across 10 random seeds and found that model ranking was broadly stable, with XGBoost remaining consistently among the best-performing models, although its advantage over TabPFN and Naive Bayes was modest in some resamples. In addition, the selected feature subsets showed similar performance on an untouched hold-out test set, providing further support that the identified parameters captured meaningful discriminatory information. However, these procedures still represent internal validation only, and confirmation in larger independent cohorts will be necessary to establish the robustness and generalizability of the findings. Although an untouched hold-out test set was reserved for final evaluation, hyperparameter tuning was not embedded within a fully nested cross-validation framework. This design allowed us to derive a single optimized model for feature ranking and SHAP-based interpretation, but some optimistic bias in model selection cannot be fully excluded.

For interpretability, SHAP was chosen over alternatives such as permutation importance due to its theoretical guarantees of consistency and local accuracy, allowing importance scores to be interpreted at both the population and individual levels [82]. To reduce the effect of multicollinearity on SHAP attribution, features with Pearson correlation coefficients above 0.9 were grouped before SHAP computation. We selected this threshold deliberately to merge only near-redundant features while preserving as much individual-feature interpretability as possible. This approach improved the stability and clinical readability of the importance rankings, but it also reduced resolution within each correlation cluster, such that the contribution of any single feature within a grouped set cannot be interpreted independently from the others. In addition, SHAP explanations remain dependent on the training data distribution on which the model was learned. Accordingly, the grouped SHAP results should be interpreted as model-dependent importance estimates within this cohort, rather than as invariant measures of feature relevance.

### Interpretation of selected gait parameters for PD and DBS

The feature selection process yielded two distinct sets: for PD classification, parameters were primarily related to medio-lateral stability deficits, bilateral coordination, and temporal control. While some prior studies identified variability as the most discriminative marker for PD [52, 56]. others highlighted asymmetry and temporal parameters as equally important [54]. For DBS classification, the retained features emphasized medio-lateral and anteroposterior stability and cross-limb coordination. Notably, several coordination features derived from CRP were consistently retained. Although less frequently emphasized in earlier gait studies, CRP has been proposed as a sensitive marker of impaired interlimb coupling in PD, which aligns with its prominence in our feature selection [83]. Although group-level comparisons showed partial improvements in temporal variability measures after DBS, these features were not retained by the classification models, suggesting that their responsiveness is inconsistent across individuals and less reliable as markers of stimulation effects.

The F1 scores for the final feature subsets were lower on the validation set than in training, reflecting a degree of overfitting. Across repeated cross-validation folds, performance showed moderate variability, with PRE vs. POST classification yielding relatively consistent results compared to the larger fluctuations observed in HC vs. PRE-DBS. This pattern suggests that while the selected features contributed meaningfully to model discrimination, their stability across samples is only partial and limited by the dataset size. Nevertheless, the convergence of statistical group differences, model performance, and biomechanical plausibility supports the credibility of the identified features.

### Overlap between the most affected gait parameters

Five gait parameters, step width asymmetry, step width variability, coordination between the left arm and right leg, coordination between the right arm and left leg, and the anteroposterior margin of stability, emerged as critical overlapping indicators of PD-related impairments and DBS responsiveness. All five shifted toward healthy control values after DBS, indicating improvements in medio-lateral stability, interlimb coordination, and forward–backward balance.

From a neurophysiological perspective, bilateral coordination deficits in PD arise from basal ganglia dysfunction, which disrupts motor planning, sequencing, and the timing of arm-leg movements [84]. Optimized bilateral DBS stimulation may partially restore these functions by modulating basal ganglia-cortical and brainstem pathways, thereby improving rhythmicity and promoting more symmetrical movement patterns [85]. Similarly, elevated step width variability and asymmetry may reflect impaired internal rhythm and timing control, resulting in inconsistent foot placement and unstable gait [86]. This interpretation is consistent with models of PD gait showing that deficits in pace, rhythm, variability, and asymmetry are key hallmarks of impaired locomotor control.

Interestingly, in our study, the mean values of basic spatiotemporal parameters did not emerge as important in either classification task. Although previous studies have reported mean-based parameters, such as step length or stance duration, as discriminative [39–44]. In our dataset, these measures were not retained after feature selection, possibly because averaging could reduce subtle but consistent gait abnormalities over a prolonged 10-minute continuous walking protocol. In contrast, variability- and asymmetry-based measures, which accumulate diagnostic value over longer recordings, provide a more robust representation of intrinsic PD pathology and DBS-treatment-associated improvements.

As a group, these five features offer a compact, physiologically grounded set of candidate gait markers that may support objective evaluation of gait impairment and gait-related treatment-associated changes in DBS-treated PD. Their relevance should be understood specifically within the gait domain, rather than as a measure of the overall clinical impact of DBS on patients or as predictors of overall DBS outcome. Their combined sensitivity to both disease-related deficits and postoperative gait changes suggests that they could be incorporated into standardized gait assessment protocols to support more precise gait-focused treatment evaluation, personalized rehabilitation planning, and longitudinal monitoring of clinically relevant changes in mobility.

### Limitations

This study has several limitations. First, the relatively small sample size limits statistical power, increases the risk of model overfitting, and may reduce the stability of feature-importance rankings across samples. Although repeated cross-validation and evaluation on an untouched hold-out test set improved confidence in model stability, these procedures still represent internal validation and cannot replace independent external replication. In addition, demographic imbalance between groups, including possible differences in sex distribution, may have acted as a residual confounder in the PRE versus control comparisons, as matching was performed on age only. Although this concern is less relevant for the paired PRE–POST analyses, sex-related differences may still influence heterogeneity of gait characteristics and limit generalizability. The parameter-wise statistical analyses should also be interpreted cautiously: although we now report FDR-adjusted q-values together with effect sizes and confidence intervals, the correlation structure among gait parameters and the limited cohort size constrain the robustness of univariate inference. We therefore regard these analyses as complementary to the machine-learning and SHAP-based feature selection, rather than as independent confirmation of candidate gait markers. Second, the study relied on a previously collected single-cohort dataset. Although appropriate for the present reanalysis, this limits novelty at the dataset level and leaves open the possibility of cohort-specific patterns. Third, post-operative gait was assessed only in the combined ON-medication and ON-stimulation condition under optimized bilateral DBS settings. As a result, the PRE–POST comparison does not allow the independent effects of stimulation to be disentangled from medication state or other post-surgical factors. At the same time, this treatment condition reflects the clinically relevant state in which DBS outcomes are typically evaluated in practice. Larger studies with independent cohorts and assessments across multiple treatment states will be necessary to confirm the robustness, specificity, and generalizability of the identified candidate markers.

## Conclusion

This study provides an interpretable machine-learning framework for identifying gait parameters that characterize PD-related gait impairment and are sensitive to postoperative treatment-associated gait changes in DBS-treated PD. By combining statistical comparisons with model-based feature selection and SHAP-based interpretation, we identified a compact set of gait parameters that distinguished PD from healthy gait and captured clinically relevant gait-domain changes between pre- and post-operative states. The consistent emergence of step width metrics, interlimb coordination measures, and the anteroposterior margin of stability underscores their potential importance in both disease-related gait impairment and gait-related treatment response. These findings suggest that a small, physiologically grounded set of gait parameters may serve as candidate markers for evaluating gait-related treatment-associated changes and tracking disease-related gait impairment in PD. With validation in larger independent cohorts, these candidate markers could be incorporated into clinical gait assessment and monitoring pipelines, supporting more objective gait-focused evaluation and individualized rehabilitation strategies.

## Supplementary Material

Below is the link to the electronic supplementary material.

Supplementary Material 1.

Supplementary Material 2.

Supplementary Material 3.

Supplementary Material 4.

Supplementary Material 5.

## Data Availability

Please contact the corresponding author for requests regarding data sharing and collaboration.
