# Supplementary figures and images for: Using explainable AI to identify disease-relevant and deep brain stimulation treatment-sensitive gait features in Parkinson’s disease
Source: J Neuroeng Rehabil. 2026 Apr 27;23:189. doi: 10.1186/s12984-026-01997-6 (PMC13262400; doi:10.1186/s12984-026-01997-6)

**Supp. Figure 1: Model comparison with repeated cross-validation**

**
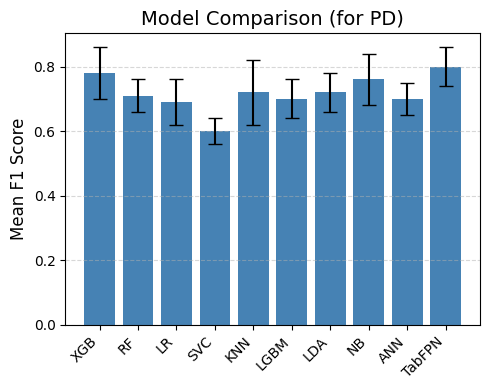
**

**
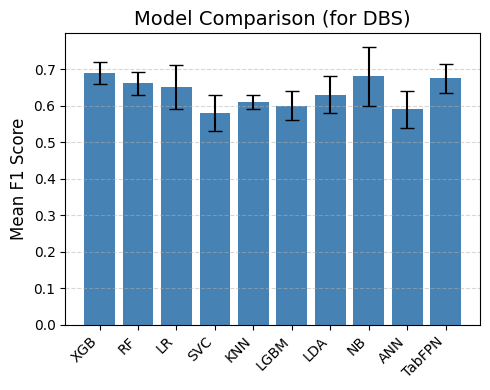
**

Supplement: Supplementary file 1 — Supplementary Material 1. [file 12984_2026_1997_MOESM1_ESM.docx]
