# Supplementary material for: Using explainable AI to identify disease-relevant and deep brain stimulation treatment-sensitive gait features in Parkinson’s disease
Source: J Neuroeng Rehabil. 2026 Apr 27;23:189. doi: 10.1186/s12984-026-01997-6 (PMC13262400; doi:10.1186/s12984-026-01997-6)
Supplement: Supplementary file 3 — Supplementary Material 3. [file 12984_2026_1997_MOESM3_ESM.docx]

**Supp. Table 1: FDR, effectSize, and CI of the parameters comparison**

Parameter-wise statistical comparisons for PRE vs. healthy controls and PRE vs. POST. For each gait parameter, the table reports the raw p-value, Benjamini–Hochberg FDR-adjusted q-value, effect size, and 95% confidence interval.

| **Gait Parameters** | **p-value**  **(pre-HC)** | **q-value (FDR)**  **(pre-HC)** | **Effect Size**  **(pre-HC)** | **CI_Low**  **(pre-HC)** | **CI High**  **(pre-HC)** |
| --- | --- | --- | --- | --- | --- |
| **Cadence (steps/min)** | 0.052 | 0.107 | -0.416 | -0.882 | -0.010 |
| **DLST (s)** | 0.408 | 0.510 | 0.176 | -0.226 | 0.605 |
| **StrideT (s)** | 0.060 | 0.110 | 0.403 | -0.003 | 0.869 |
| **StanceT (s)** | 0.112 | 0.180 | 0.340 | -0.078 | 0.800 |
| **SwingT (s)** | **0.028** | 0.062 | 0.471 | 0.070 | 0.962 |
| **StepL (cm)** | 0.710 | 0.776 | 0.079 | -0.333 | 0.516 |
| **StepT (s)** | 0.058 | 0.110 | 0.406 | -0.020 | 0.871 |
| **StepW (cm)** | 0.484 | 0.585 | -0.148 | -0.567 | 0.267 |
| **StrideL (cm)** | 0.705 | 0.776 | 0.080 | -0.322 | 0.506 |
| **WalkingSpeed (cm/s)** | 0.395 | 0.510 | -0.181 | -0.627 | 0.247 |
| **StepL Asy** | **0.002** | **0.009** | 0.680 | 0.268 | 1.159 |
| **StepW Asy** | **0.002** | **0.009** | 0.681 | 0.273 | 1.135 |
| **StepT Asy** | **0.015** | **0.042** | 0.526 | 0.101 | 1.056 |
| **StanceT Asy** | 0.076 | 0.133 | 0.380 | -0.034 | 0.775 |
| **SwingT Asy** | 0.154 | 0.224 | 0.304 | -0.104 | 0.674 |
| **PCI_LeftvsRight** | 0.793 | 0.837 | 0.056 | -0.356 | 0.484 |
| **PCI_ShortvsLong** | 0.813 | 0.837 | 0.050 | -0.364 | 0.507 |
| **CRP_Leg&Leg** | **0.016** | **0.042** | 0.521 | 0.127 | 0.950 |
| **CRP_arm&arm** | **0.010** | **0.033** | 0.555 | 0.162 | 0.958 |
| **CRP_Rarm&Lleg** | **<0.001** | **0.002** | 0.842 | 0.537 | 1.207 |
| **CRP_Larm&Rleg** | **<0.001** | **0.002** | 0.837 | 0.546 | 1.178 |
| **CRP_Rarm&Rleg** | **0.026** | 0.060 | 0.480 | 0.180 | 0.755 |
| **CRP_Larm&Lleg** | **0.025** | 0.060 | 0.481 | 0.174 | 0.757 |
| **MOS_AP** | **0.005** | **0.021** | -0.603 | -0.944 | -0.252 |
| **MOS_ML** | 0.227 | 0.318 | -0.257 | -0.694 | 0.158 |
| **Cadence_Var (% CV)** | 0.867 | 0.867 | 0.036 | -0.328 | 0.546 |
| **DLST_Var (% CV)** | **0.010** | **0.033** | 0.555 | 0.120 | 1.181 |
| **StrideT_Var (% CV)** | 0.625 | 0.730 | 0.104 | -0.262 | 0.683 |
| **StanceT_Var (% CV)** | 0.336 | 0.452 | 0.204 | -0.184 | 0.801 |
| **SwingT_Var (% CV)** | **0.004** | **0.017** | 0.626 | 0.175 | 1.291 |
| **StepL_Var (% CV)** | **<0.001** | **0.001** | 0.970 | 0.581 | 1.423 |
| **StepT_Var (% CV)** | 0.141 | 0.215 | 0.314 | -0.089 | 0.995 |
| **StepW_Var (% CV)** | **<0.001** | **0.003** | 0.783 | 0.401 | 1.215 |
| **StrideL_Var (% CV)** | **0.001** | **0.005** | 0.741 | 0.316 | 1.200 |
| **WalkingSpeed_Var (% CV)** | 0.113 | 0.180 | 0.338 | -0.072 | 0.662 |

| **Gait Parameters** | **p-value**  **(pre-post)** | **q-value (FDR)**  **(pre-post)** | **Effect Size**  **(pre-post)** | **CI_Low**  **(pre-post)** | **CI High**  **(pre-post)** |
| --- | --- | --- | --- | --- | --- |
| **Cadence (steps/min)** | **0.004** | **0.025** | 0.437 | 0.156 | 0.789 |
| **DLST (s)** | 0.255 | 0.331 | -0.164 | -0.505 | 0.111 |
| **StrideT (s)** | **0.004** | **0.025** | -0.439 | -0.772 | -0.162 |
| **StanceT (s)** | **0.014** | 0.056 | -0.365 | -0.701 | -0.089 |
| **SwingT (s)** | **0.005** | **0.026** | -0.419 | -0.740 | -0.143 |
| **StepL (cm)** | 0.214 | 0.299 | -0.180 | -0.486 | 0.090 |
| **StepT (s)** | **0.003** | **0.025** | -0.439 | -0.766 | -0.158 |
| **StepW (cm)** | 0.094 | 0.210 | 0.244 | -0.029 | 0.527 |
| **StrideL (cm)** | 0.196 | 0.286 | -0.187 | -0.495 | 0.093 |
| **WalkingSpeed (cm/s)** | 0.628 | 0.709 | 0.070 | -0.203 | 0.365 |
| **StepL Asy** | 0.326 | 0.407 | 0.142 | -0.139 | 0.444 |
| **StepW Asy** | **0.005** | **0.026** | -0.417 | -0.686 | -0.169 |
| **StepT Asy** | 0.087 | 0.210 | 0.250 | -0.027 | 0.562 |
| **StanceT Asy** | 0.140 | 0.238 | 0.214 | -0.064 | 0.600 |
| **SwingT Asy** | 0.128 | 0.238 | 0.221 | -0.054 | 0.556 |
| **PCI_LeftvsRight** | 0.385 | 0.449 | -0.125 | -0.400 | 0.166 |
| **PCI_ShortvsLong** | 0.380 | 0.449 | -0.127 | -0.409 | 0.168 |
| **CRP_Leg&Leg** | 0.894 | 0.920 | 0.019 | -0.261 | 0.287 |
| **CRP_arm&arm** | 1.000 | 1.000 | 0.000 | -0.306 | 0.261 |
| **CRP_Rarm&Lleg** | **0.003** | **0.025** | -0.455 | -0.657 | -0.261 |
| **CRP_Larm&Rleg** | **0.003** | **0.025** | -0.456 | -0.650 | -0.266 |
| **CRP_Rarm&Rleg** | **0.018** | 0.056 | -0.351 | -0.525 | -0.174 |
| **CRP_Larm&Lleg** | **0.018** | 0.056 | -0.351 | -0.521 | -0.170 |
| **MOS_AP** | 0.143 | 0.238 | 0.215 | -0.061 | 0.484 |
| **MOS_ML** | 0.810 | 0.865 | -0.035 | -0.342 | 0.240 |
| **Cadence_Var (% CV)** | 0.179 | 0.272 | 0.195 | -0.085 | 0.458 |
| **DLST_Var (% CV)** | 0.067 | 0.180 | 0.268 | 0.002 | 0.498 |
| **StrideT_Var (% CV)** | 0.141 | 0.238 | 0.214 | -0.068 | 0.454 |
| **StanceT_Var (% CV)** | **0.042** | 0.121 | 0.299 | 0.032 | 0.558 |
| **SwingT_Var (% CV)** | 0.096 | 0.210 | 0.243 | -0.035 | 0.490 |
| **StepL_Var (% CV)** | 0.177 | 0.272 | 0.196 | -0.077 | 0.506 |
| **StepT_Var (% CV)** | 0.134 | 0.238 | 0.218 | -0.059 | 0.444 |
| **StepW_Var (% CV)** | **0.017** | 0.056 | -0.352 | -0.630 | -0.072 |
| **StrideL_Var (% CV)** | 0.222 | 0.299 | 0.177 | -0.105 | 0.487 |
| **WalkingSpeed_Var (% CV)** | 0.816 | 0.865 | -0.034 | -0.262 | 0.351 |
