# Supplementary material for: Using explainable AI to identify disease-relevant and deep brain stimulation treatment-sensitive gait features in Parkinson’s disease
Source: J Neuroeng Rehabil. 2026 Apr 27;23:189. doi: 10.1186/s12984-026-01997-6 (PMC13262400; doi:10.1186/s12984-026-01997-6)
Supplement: Supplementary file 5 — Supplementary Material 5. [file 12984_2026_1997_MOESM5_ESM.docx]

**Supp. Table 3: Parameter Clusters**

| **Groups** | **Parameters in the group** |
| --- | --- |
| **1** | 'SwingT Asy', 'StanceT Asy' |
| **2** | 'CRP arm&arm', 'CRP Leg&Leg' |
| **3** | 'CRP Larm&Rleg', 'CRP Rarm&Lleg' |
| **4** | 'CRP Larm&Lleg', 'CRP Rarm&Rleg' |
| **5** | 'PCI ShortvsLong', 'PCI LeftvsRight' |
| **6** | 'StrideT', 'Cadence', 'StepT', 'StanceT', 'DLST' |
| **7** | 'StrideT Var', 'StanceT Var', 'StepT Var', 'Cadence Var' |
| **8** | 'StepL', 'StrideL' |
| **9** | 'StepW Var', 'StepW Asy' |
| **10** | 'StrideL Var', 'StepL Var' |
| **11** | 'Walking Speed' |
| **12** | 'StepW' |
| **13** | 'MOS ML' |
| **14** | 'MOS AP' |
| **15** | 'Walking Speed Var' |
| **16** | 'StepT Asy' |
| **17** | 'DLS Var' |
| **18** | 'SwingT Var' |
| **19** | 'StepL Asy' |
| **20** | 'SwingT' |
